# 

**Published:** 1908-04

**Authors:** 


					﻿The Cause and Prevention of Rape.
Vol. XXIII.
APRIL, 1908.
No. 10. '
T E X AS
MEDICAL I
__	_ _____	V -B.	'
JOURNAL
“RED BACK”
■ PUBLISHED AT AUSTIN, TEXAS, BY
DANIEL, M. D.,	-	- Proprietor
JNTED BY THE VON BOECKMANN-JONES CO.
Subscription, $1.00 a Year, in Advance. -	-	- Single Copies, 10 Cents
Entered at the Postoffice at Austin, Texas, as Second-class Mail Matter
				

